# Central Dental Association of Northern New Jersey

**Published:** 1885-03

**Authors:** 


### CENTRAL DENTAL ASSOCIATION OF NORTHERN NEW JERSEY.

A regular meeting of the Society was held at the residence of Dr. D. C. McNaughton, Jersey City, on the evening of Monday, Dec. 15, 1884. After the transaction of the usual routine business a paper was read by Dr. C. A. Marvin, upon the "Treatment of Defective Teeth During Pregnancy." (See page 115.)

#### DISCUSSION.

*Dr. W. H. Atkinson*—There is very little to be said respecting the sound sense of the mode of management prescribed in the paper, but as to the facts that it postulates there may be room for discussion.

There are no statistics that can be relied upon concerning the increased or the lessened sensibility of teeth in the condition spoken of, but good sense and observation tell us that when the functions that are natural to the body are well performed, we have the highest type of health.

The paper refers briefly to the fact that some pregnant women are able to bear more pain than others, or than they themselves could do in a non-pregnant state. I know that to be a fact. But as to general principles, and our ability to determine whether irritation depends upon or coincides with this state, we must be better diagnosticians than most of us are, to tell exactly why that irritation exists. I have practiced obstetrics, and I have known of other men practicing obstetrics, who were unable to determine whether or not a woman was pregnant, when she was seven months advanced. So it is our want of ability to understand exactly what is going on in the system, that prevents us from being able to determine what is the extent of any special kind of irritation that we may see in the patient, when that irritation is greater than it was under other circumstances. The best results I have seen from the use of cocaine was in the case of a pregnant woman—at least I supposed she was pregnant. By applying cocaine I succeeded in filling four very sensitive cavities in the buccal walls of the right superior bicuspids and molars. I used a four per cent. solution, without putting on a rubber dam, and the patient thought I was only getting ready to put it on when the burring was all done. I have had the same impression that Dr. Marvin expresses when he says that it is best to temporize, and I think in that case it was wise to do so. The right superior bicuspids and cuspids were in a very uncomfortable condition; one of them was pulpless, and the gums were swollen and inflamed, so that when the lady ate on that side they bled. It took several days to reduce that tumid condition. My mode of procedure was to dry the gums as well as I could with bibulous paper, and then cook them with the saturated solution of salicylic acid and alcohol, so that they would be whitened. I think that was repeated every other day for four or five sittings. I succeeded in temporarily filling some of the teeth that were not so tender. I was dressing them, as I often have done before, with oil of cloves, which is now being represented by what is called eugenol. It is an oxygenated oil of cloves. When I got them where I could handle them well I attempted to excavate, and the lady complained of the pain. I said I had just learned about a new article which was said to be serviceable in those cases, and I would try it. She asked me to wait another day. I did wait, and finally applied cocaine with the result I have stated.

I have too much respect for the observation of Dr. Marvin, and for his goodness of heart, to question any of the statements that he makes, and I only offer these remarks as a suggestion that we do not reach causes. We get at antecedents and sequences. It has been the difficulty of my life to get rid of the false teaching that I received in my instruction as a medical man. Things were called causes simply because they had preceded the conditions that were present. If I were to deal with causes at all, I should keep it in the plural, and not say *cause*, for the real cause none of us knows anything about.

I think civilization is chargeable with the deterioration of the teeth and of the race, and a false civilization at that; a false sentiment which respects and defers to the idea that it is a shame for a woman to be pregnant. If women were taught that this is their glory, they would carry their burden joyously, and with a face flushed with pride instead of shame. It is that false sentiment that deteriorates women physically and morally.

The manner in which women clothe themselves has been a very great mistake. There is a constant crowding of the contents of the pelvic region. My first practice was in an agricultural district, and some of the finest children I ever saw were produced there by mothers who worked up to the day of their confinement. Some of them worked during the day of their confinement, waiting upon themselves and attending to their household duties right along.

If we are really interested in our patients, there is not one of us so bad but that he will respect them, and feel when they flinch. Any dentist who glories in extracting teeth when a patient shrinks I would expel from the profession, and make it impossible for him to exercise unkindness and severity upon those who were so unfortunate as to fall into his power. What the doctor says is very true, that many noble women have suffered untold agony on account of their children. Nothing but moral strength can enable them to bear up under their terrible burden. When we deal with this question to the exclusion of its moral side, I feel a little tender, and a little angry; and when I remember to whom we owe our false teachings, I can hardly enter into a discussion without the latter feeling; for the older I get the more I feel like departing from the instruction I received in my early life.

If I have anything to say to a young man it is this: that he let his relations with his patients be as they would be with his mother or his sister, whether pregnant or not. I endorse most heartily my beloved brother's statement, when he says that we should remember that there is such a thing as exalted sensibility in women, and when they shrink we should give of our magnetism; not in any namby-pamby or demoniac way, but in its diviner and more fraternal and childlike manifestations—it is the heart that controls, not the head. The head dies. Give me the man with the big heart, and even though he is an ignoramus at the beginning, there is hope for him. And I don't care how big a man's head is, or how well cultivated his intellect, if he is heartless, for God's sake pray that he may never enter such a calling as is ours.

*Dr. C. S. Stockton*—This subject has been so eloquently and wisely, and with such sound common sense discussed in the paper, that there does not really seem to be anything more to say upon the subject. I am sure I have been repaid for my journey here to-night, in hearing this paper.

There is a false civilization, and a false state of society, perhaps; but we cannot help but admire the modesty of a pregnant woman who shields her condition, and who presents herself to the world in that state with as little show as may be. It may be a mock sentiment that causes women to present themselves or to shield themselves when in that condition, but there is not a man among us all, I think, who will not say that she is a true woman, and possessed of a modesty that becomes her. This is a condition of which a very wise gentleman once said, that if the man bore the children there would be but three persons in a family. And that is true; and we ought to have all the consideration, and all the patience, and all the love that our hearts can extend to women in this condition, who may come into our hands. We do not know a very great deal about it, for, as Dr. Atkinson has said, there are no statistics on the subject, but the fact seems to be that all women are more sensitive when in that state, and more teeth are lost than there would be if women did not bear children. The old saying, "for every child a tooth," grows out of the fact that women's teeth do decay more rapidly during pregnancy than they do at other times. The question resolves itself largely into a matter of hygiene, which I freely confess I know but little about; I wish I knew a great deal more. I remember an instance in which the wife of a physician was in my hands during her first pregnancy. Her teeth decayed very rapidly, superinduced by her condition. I could only do temporary work, as I do in all such cases. I consulted with her husband, and I said to him, "the next time your wife is in such a condition take better care of her health. You know what she has to support, and what she has to supply, and you can by your wise and judicious treatment save her a great deal of trouble." During the next two pregnancies he did so, and it was a marvel to note the contrast in her health, and also to see the difference in the teeth of those two children. They do not look as if they belonged to the same family.

*Dr. Meeker*—I have listened with a great deal of pleasure to Dr. Marvin's paper, and also to the remarks of Dr. Atkinson. Both of them have mentioned one fact, the exercise of mesmeric force or magnetic force on the part of the operator, and I judge from Dr. Marvin's long practice and his remarks in the paper, that he is in the habit of exercising that force. I would like to hear from him a little more concerning it, and whether he notices any difference in his own nervous exhaustion when operating for patients of different nervous temperaments.

*Dr. Marvin*—Yes, I certainly do. I have not any formula to explain the exercising of this force, and I do not claim any special potency in that direction. I simply speak of the fact, which was first brought especially to my notice by a remark made by one of my patients, a nervous young woman, not married, and therefore outside of the range of the discussion to-night; but she was a very nervous woman. She said this to me: "I do not know why it is, but whenever I am in your chair and under your hands I seem to get a kind of sustaining force from you that I never have at any other time." I cannot analyze it; it is one of those mysterious things that are inexplicable, and yet we so often see the results of this influence that we cannot deny its existence. My own feeling at such time is one of as perfect nerve balance as I am ever in; a calm, quiet state, so that when there is any exhibition or manifestation of pain I use no harshness, no controlling words of severity, but maintain an even, quiet bearing toward my patient; at the same time I unconsciously seem to be giving off from myself a sort of power or influence which weakens me, so that I am completely exhausted when I am done. Perhaps you can explain it; I cannot. When I am engaged in a long operation I seem to be under a high tension. I feel that there is a sort of emanation of force, accompanied by a degree of tension which I cannot explain, but all the while there is exercised an unseen, unexplained influence upon my patient. At first I did not do it with the expectation of accomplishing anything by it; it was simply from intuition; I did not think anything about it particularly. Then I began to look at the subject a little more, and I found that the more quiet, gentle, calm and deliberate I was, the more influence seemed to be exercised over my patients, and they seemed to be enabled more easily to endure painful operations. They must see no excitement in your face; all about your manner must indicate perfect poise; you are gentle, yet firm, with a consciousness that you are doing the right thing, and you feel that it is proper for them to endure it. Your firmness and calmness impresses your patients; they are taking from you some force that I cannot explain, but you feel it when you get through; you feel like going to bed.

I want to say that I do most heartily thank Dr. Atkinson for the expression he has made of a sentiment that touches me. I do not know that I ever feel a more tender sentiment in my mind, or a more tender feeling in my heart, than I do for a woman when she is in that condition. It is a perfectly proper and holy feeling, if there ever was one. A man would be worse than a beast had he any but the right sort of feeling for a woman who is pregnant, no matter what he might be at another time. There is a peculiar feeling of tenderness that comes over me at such a time for a woman in that condition, which I cannot express. I cannot understand how a man could have any other than the most pure and tender feeling toward a woman who is passing through that period of delicacy and trial, which he can never experience. There is something that appeals to the best instincts of man's nature when a woman comes into his hands to have an operation performed, when she is in that delicate condition. What I have stated in the paper was not based upon statistics; I do not know that there are any; it is simply drawn from my own experience. I have noticed a great difference in women in respect to dental operations. Some can bear them better than others; but the same women seem to be more sensitive at such times than they were before. My experience has been of the nature that led me to conclude, long ago, that when pregnant women come into my hands to have their teeth attended to, or women I suspect to be pregnant, it was better to do only temporary work, and I say to them: "Madam, I think the best thing to do will be to fill your teeth temporarily; for the present I will put them in a safe condition, and by and by you will be better able to have the permanent work performed." If the woman is acute she will understand it, and if she does not it is no matter. It is said gently, of course, and she usually understands and appreciates it. I think that course better for the woman, better for her teeth, and better for the dentist, aside from the matter of expediency. If the dentist shows himself regardful of a woman in such a condition, he will make a ten times better impression upon her than he would by showing himself regardful of her feelings in any other circumstances. There is something about that evidence of consideration that goes down deep. It is just the same as with a husband who shows his consideration at such a time; it is better than forty manifestations of tender feeling at any other time. So if a dentist shows himself tender and regardful of women who are in such a condition, he will do himself a great benefit, as well as do the right thing by them.

I wish I could say something more regarding the matter of mesmerism, or magnetic force. It is something that we are occasionally inclined to ridicule, and we cannot explain it, but it is a fact. The first requisite is to keep yourself well poised. If a man is shaky himself, he cannot exercise an influence over another. If he keeps his head right, his purpose high, and his knowledge of what he is doing true, he can exercise an influence on his patient that is marvelous. I have seen it done many a time, in a way that was surprising. But you have to pay for it afterwards ; you find yourself quite exhausted.

*Dr. Meeker*—I am glad to hear Dr. Marvin's explanation. I remember that a few years ago I read a paper before our State society on that subject, and I was so completely squelched by our good friend Dr. Atkinson, that I have always been afraid to speak about it again. I think some of my brother members also gave me a severe talking to. Still it has been a very interesting subject to me, simply because I have frequently noticed the wonderful effects of this exercise of mesmeric or magnetic force. I do not know whether the same fact applies to other people, but I know that I have exercised that influence, and I have felt very bad, at times, after a long sitting, so much so that all the ambition was taken out of me, and I was unable to work. On some patients I have a very strong influence ; but for the past few years I have tried to avoid the exercise of any such influence, even to calm people who were very nervous, because the strain upon me was too great. I feel satisfied, from my own experience and from the statements of others with whom I have talked upon the subject, that there is such an influence of the operator upon his patient, some possessing it in a greater degree than others.

*Dr. G. S. Meigs*—On the subject of personal magnetism I have in my mind's eye two instances where the phenomena are directly opposite. I have a young friend in Philadelphia who is a painter, quite an artist ; she is not married, is in poor health, and has enough ambition for two or three people, and if she is hurried by her customers with her work she is apt to do in a week what should require two or three weeks' time, and then, as a consequence, go to bed for four or five days. That girl has a wonderful influence over a crying child. She rarely fails, by taking a child in her arms and talking to it, to stop its crying very quickly. I have asked her how she does it, and she says, "I mesmerize them ; but I do not like to do it, because it is exhausting to me." She is a girl who is without any special force of character at all, and you would not dream that she possessed this power. In opposition to that case I have a friend, a physician in New York, who is really one of the brightest and best educated men to be found anywhere ; what he knows he can express in the quickest and clearest manner, and he is an accurate and elegant writer, but when a patient comes to him there is not a particle of sympathy between them, and this unsympathetic manner is so marked and disagreeable that he does not succeed very well in business.

*Dr. Watkins*—I want to express my thanks for every word that Dr. Marvin has said in regard to the magnetic influence of the dentist over his patient, for I think it is wholly true. I believe it is also true that the patient has a very marked influence on the dentist. I have noticed a great many cases in which the patient was nerved up, and seemed to be strengthened by the operation, and I have wilted ; and I have seen other cases where it seemed as though I could work a week without fatigue, and my patient would lag and be completely used up. I have seen patients in a couple of hours' sitting all fagged out ; and in other cases after a two hours' sitting I would be fagged out, and the patient would be fresh.

In regard to working upon children, I have never known why it is that I have been able to get along with children with so little trouble. I do not know now whether or not it is due to magnetic influence, but I always make a practice of being very firm, and yet gentle, with children, and I think my children patients are my best ones ; they usually behave beautifully.

Dr. Meigs spoke of personal magnetism. I was called the other evening to see a patient, the wife of a prominent dentist, who has been a terrible sufferer. When I arrived at her house I found her completely mesmerized ; she did not recognize me for some time. An old gentleman had simply taken here hand in his and rubbed her forehead, and she had become so thoroughly mesmerized as to be completely under his control, and did not know anything. I suppose I was in the house twenty minutes before I was able to do anything to give her relief. After the mesmeric influence passed away the pain returned. I then proceeded to apply remedies.

In regard to cases of pregnancy, I think that is just where cocaine is destined to be a grand remedy. The other day I had a case of a person who is not very intelligent. Perhaps some of you have noticed that in persons who are lacking a little in intellect, the teeth are very sensitive and the saliva stringy. I put the rubber dam on a superior cuspid and two biscuspids, examined the cavities and found them quite large. The cuspid was so sensitive that she could not bear to have me touch it at all. I put some cocaine in the tooth, and went to work on the others. After a while, when I happened to think of that tooth, I returned to it. The cotton conveying the cocaine was entirely dry. I put in my excavator and began to cut away, without causing a particle of pain. After excavating a minute or two a little sensitiveness was developed, and I put in more cocaine and went to work at the other teeth. After completing them I went back to the cuspid, and finished excavating it without any further trouble.

Dr. Marvin also spoke of the use of gutta-percha as a temporary stopping. I have found that pink gutta-percha, that is the common base-plate gutta-percha, is an excellent article for that purpose. I have heard a gentlemen who is here make the remark that he had noticed that the pink wore very much better than other gutta-perchas; that is my experience. I have used it a great deal; in fact, have gotten out of the way of putting in the other gutta-percha at all, unless it is simply a temporary operation, for it seems to wear so poorly. With pink guttapercha I can make a filling that will be permanent. I saw a case to-day where I had, three and a half years ago, filled with pink gutta-percha a cavity in the posterior surface of a lower second bicuspid, including fully one-half of the crown surface, there being a good articulation, and that filling to-day shows scarcely any wear at all. The patient is a gentleman twenty-four years of age. The pink gutta-percha does not appear to decompose in the mouth like the other kinds, that seem to get soft and spongy and almost rotten on the surface, so that you can put in an instrument and pick them all to pieces. It seems to become more dense, and you have quite a job to remove it. The fluids of the mouth do not seem to penetrate it as they do the other kinds, and it wears better. Seven years ago a gentleman told me that he had in his mouth a filling of that kind that had then been in twenty-six years. It is now thirty-three years since it was inserted, and it remains in, and is in such good condition that I do not think there is a dentist here who would remove it. It is in the labial surface of a lower cuspid. If any of you have not tried pink gutta-percha I wish you would do so. For cavities in the buccal surfaces of molars, or almost any surface of the wisdom teeth, it makes a capital filling.

While Dr. Atkinson was speaking of women who during pregnancy were ashamed of their condition, I could not help thinking of a journalist who published a paper in Montclair a few years ago. He was very enterprising in gathering and publishing the news of the town, and sometimes the people thought he gave rather too many items. In one of his issues he had a little squib saying there was a party at a certain house, and that there were sixteen married ladies present, thirteen of whom were in that interesting condition in which all good women love to be. It is needless to say that he was very unpopular, and that the paper died early.

*Dr. Atkinson*—One thing further in the way of a little amplification of the idea of imcompatibility. I had a patient who came under my care in the most distressing circumstances. I did a large amount of work for her, and after she had been twenty-four days under the operation her husband came to me and said: "What have you been doing to my wife? she never before has had an operation performed in her mouth without being ill for three or four weeks in consequence. Now instead of coming home and taking to her bed as she has been accustomed to do after such operations, she makes her toilet and goes out and makes visits in the highest spirits." That patient is the type of a great number that I have had, and in these cases I was not exhausted, but could work vigorously during the entire light of a long day, and do satisfactory work, and we were better and stronger when through than when we began. That smacks a little of the marvelous, I suppose, but it really is instructive to me. I call that compatibility between patient and operator. I would very much prefer to have compatible patients who coincide with me, than to have to force them to submit to my ideas and dictation, or myself succumb to their views. There will come a time when we shall understand these things, and be enabled to comprehend them in a better light, and to handle ourselves and our patients more wisely and successfully. Dr. Marvin says that a certain influence is exerted by the operator upon his patient, but what that influence is does not appear so clearly upon the face of it.

*Dr. Luckey*—We have heard a great deal about mesmerism tonight. While I do not propose to try to explain what is the force that goes out from the operator to the patient, I do know that one reason why patients can be controlled easily and thoroughly is, that on the face of the operator is stamped the fact that he has perfect confidence, and knows just what he is going to do. They see in his face knowledge and ability ; they see that he knows positively what he is about, and that will gain for any operator the confidence of his patients. But if the operator is hesitating in his answers to their questions, or vacillating and uncertain as to what should be done, he loses the confidence of those who come to him, and often fails to make a patient. I think this positive and unhesitating manner will account for the confidence with which patients sit down and submit to operations at the hands of some operators.

As to a man feeling tired, worn out, that is not so often due to the outgoing of mesmeric force as to the fact that the operator gives to his work so much intensity of application ; he concentrates his whole being in the effort to perform the operation successfully, and when he has finished it, and the tension is removed, he feels languid and depressed. That condition may be owing to the exercise of mesmeric power, but I think it is rather due to intensity of application.

The temporizing treatment of teeth in the mouths of pregnant women is an excellent thing. But I do not confine it to pregnant women; it is properly applicable to any woman, or any man, whose teeth are in a hypersensitive condition. In all such cases I think the best practice is to temporize, filling with either gutta-percha or phosphates, until the teeth are in a less sensitive condition. If you fill a tooth when it is very sensitive, it is very likely to give trouble by bringing on tooth-ache or abscess, and its attendant troubles.

